# Supplementary material for: The level of protein in the maternal murine diet modulates the facial appearance of the offspring via mTORC1 signaling
Source: Nat Commun. 2024 Mar 26;15:2367. doi: 10.1038/s41467-024-46030-3 (PMC10965948; doi:10.1038/s41467-024-46030-3)
Supplement: Supplementary file 1 — Supplementary Information [file 41467_2024_46030_MOESM1_ESM.pdf]

Supplementary Figures 1-4

For the paper titled "*The level of protein in the maternal murine diet modulates the facial appearance of the offspring via mTORC1 signaling*".

By Authors: Meng Xie[1-3], Markéta Tesařová[4], Yaakov Gershtein[5], Daniela Schnyder[6], Ruslan Deviatiiarov[7-9], Guzel Gazizova[7], Elena Shagimardanova[7,9] Tomáš Zikmund[4], Greet Kerckhofs[10-13], Evgeny Ivashkin[14,15], Dominyka Batkovskyte[1], Phillip T Newton[1,16,17], Olov Andersson[18], Kaj Fried[19], Oleg Gusev[7-9,20], Hugo Zeberg[1], Jozef Kaiser[4], Igor Adameyko[1,5*], Andrei S Chagin[1,6*]

Affiliations

1. Department of Physiology and Pharmacology, Karolinska Institutet, Stockholm, Sweden
2. Department of Biosciences and Nutrition, Karolinska Institute, Flemingsberg 14183, Sweden
3. School of Psychological and Cognitive Sciences, PKU-IDG/McGovern Institute for Brain Research, Peking University, Beijing, China.
4. Central European Institute of Technology, Brno University of Technology, Brno, Czech Republic
5. Department of Neuroimmunology, Center for Brain Research, Medical University of Vienna, 1090 Vienna, Austria.
6. Centre for Bone and Arthritis Research, Institute of Medicine, Sahlgrenska Academy at University of Gothenburg, Gothenburg, Sweden
7. Regulatory Genomics Research Center, Kazan Federal University, Kazan, Russia
8. Endocrinology Research Center, Moscow, Russia
9. Life Improvement by Future Technologies (LIFT) Center, 143025 Moscow, Russia
10. Biomechanics Lab, Institute of Mechanics, Materials, and Civil Engineering (iMMC), UCLouvain, Louvain-la-Neuve, Belgium
11. Pole of Morphology, Institute of Experimental and Clinical Research (IREC), UCLouvain, Woluwe, Belgium
12. Department of Materials Engineering, KU Leuven, Leuven, Belgium
13. Prometheus, Division for Skeletal Tissue Engineering, KU Leuven, Leuven, Belgium
14. A.N. Severtsov Institute of Ecology and Evolution, Russian Academy of Sciences, Moscow, Russia
15. Department of Developmental and Comparative Physiology, N.K. Koltsov Institute of Developmental Biology, Russian Academy of Sciences, Moscow, Russia
16. Department of Women's and Children's Health, Karolinska Institutet, Sweden.
17. Astrid Lindgren Children's hospital, Stockholm, Sweden.
18. Department of Cell and Molecular Biology, Karolinska Institutet, Stockholm, Sweden
19. Department of Neuroscience, Karolinska Institutet, Stockholm, Sweden
20. Intractable Disease Research Center, Juntendo University, Tokyo, Japan

*Corresponding authors:
Andrei S Chagin, Igor Adameyko
andrei.chagin@gu.se, igor.adameyko@meduniwien.ac.at

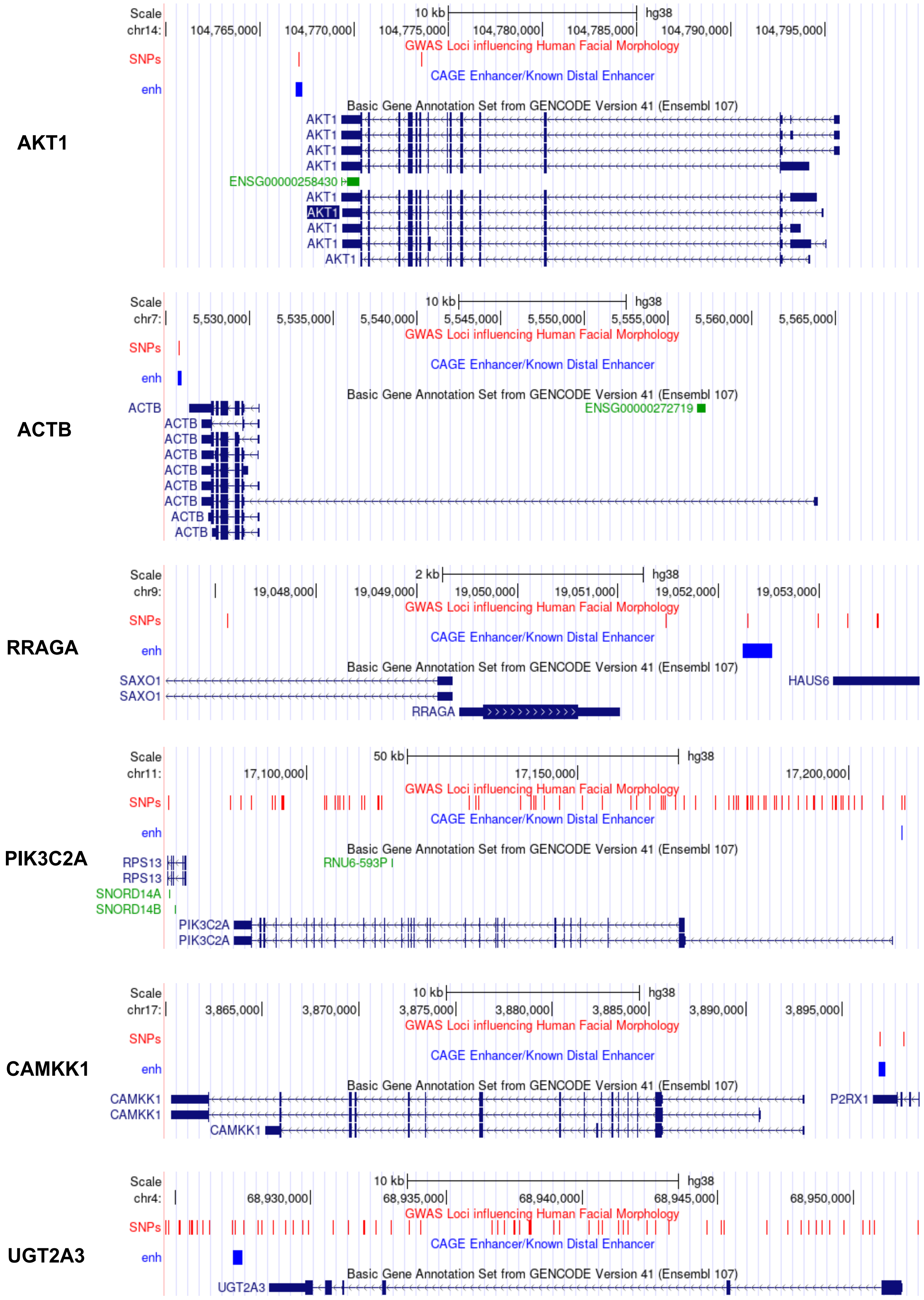

Supplementary Figure 1. Gene maps showing SNPs associated with facial appearance within the normal range and CAGE-identified active enhancers for genes associated with PI3K/mTORC1/autophagy.

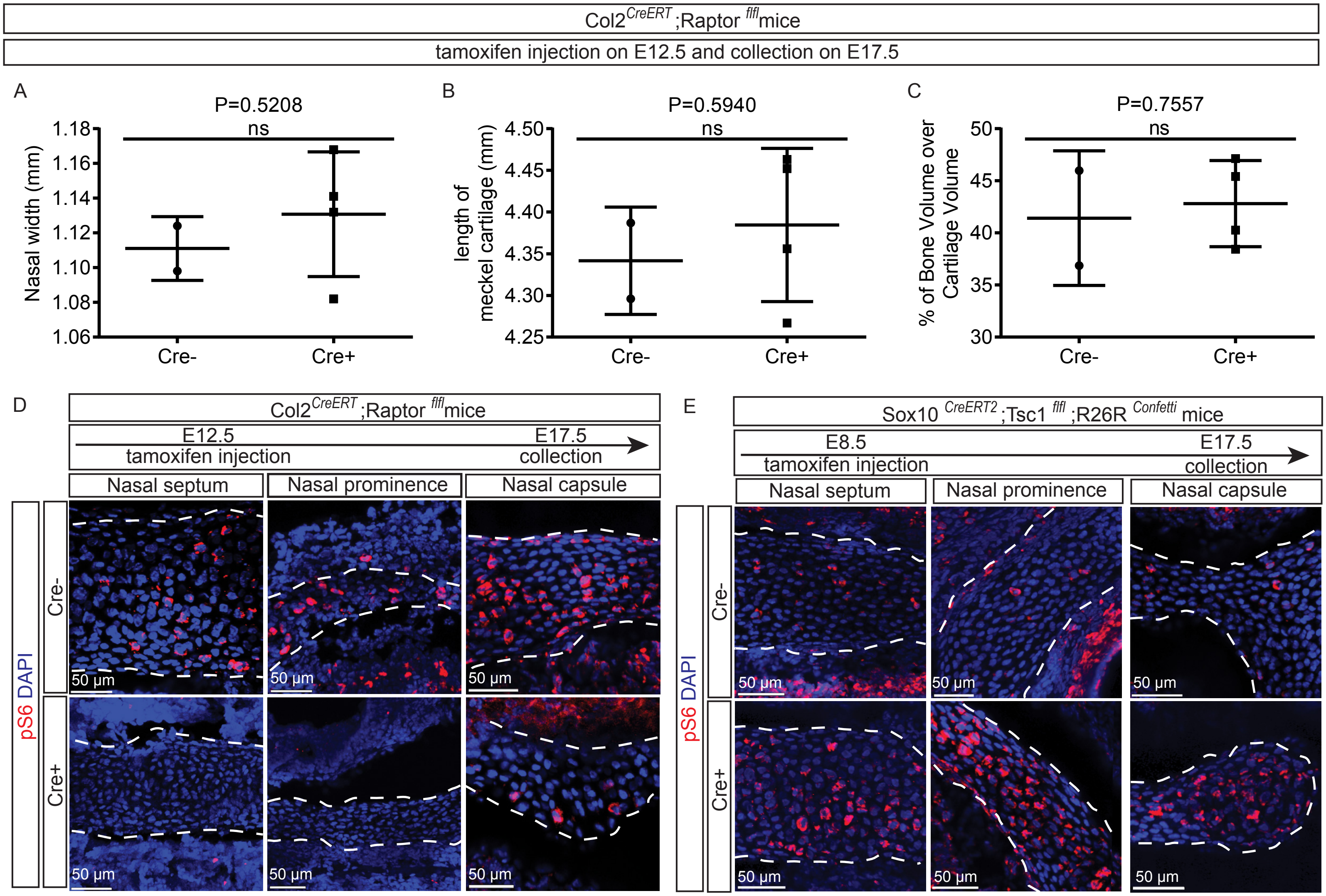

Supplementary Figure 2. *Craniofacial parameters in mice lacking Raptor gene in their chondro-progenitors and mTORC1 activity in cartilage elements*

(A-C) The size of the craniofacial skeleton of $Col2^{CreERT}$;$Raptor^{fl/fl}$ mice pulsed with tamoxifen on E12.5 was determined on E17.5. n=2 and 4 animals in (A-C). (D-E) The activity of the mTORC1 pathway in elements of the nasal cartilage of either $Col2^{CreERT}$;$Raptor^{fl/fl}$ mice (D) pulsed with tamoxifen on E12.5 or $Sox10^{CreERT2}$;$Tsc1^{fl/fl}$;$R26R^{Confetti}$ mice (E) pulsed with tamoxifen on E8.5 was assessed on the basis of the level of S6 phosphorylation. All embryos were analyzed on E17.5. The controls are designated Cre- and the knock-outs Cre+. In D and E, phosphorylated S6 is colored pink, and the nucleus is counterstained with DAPI (blue). The white dashed lines outline the cartilage. Means ± SD are presented in A-C, with individual values also indicated. Statistical analysis was performed utilizing the two-sided unpaired t-test. Source data are provided as a Source Data file.

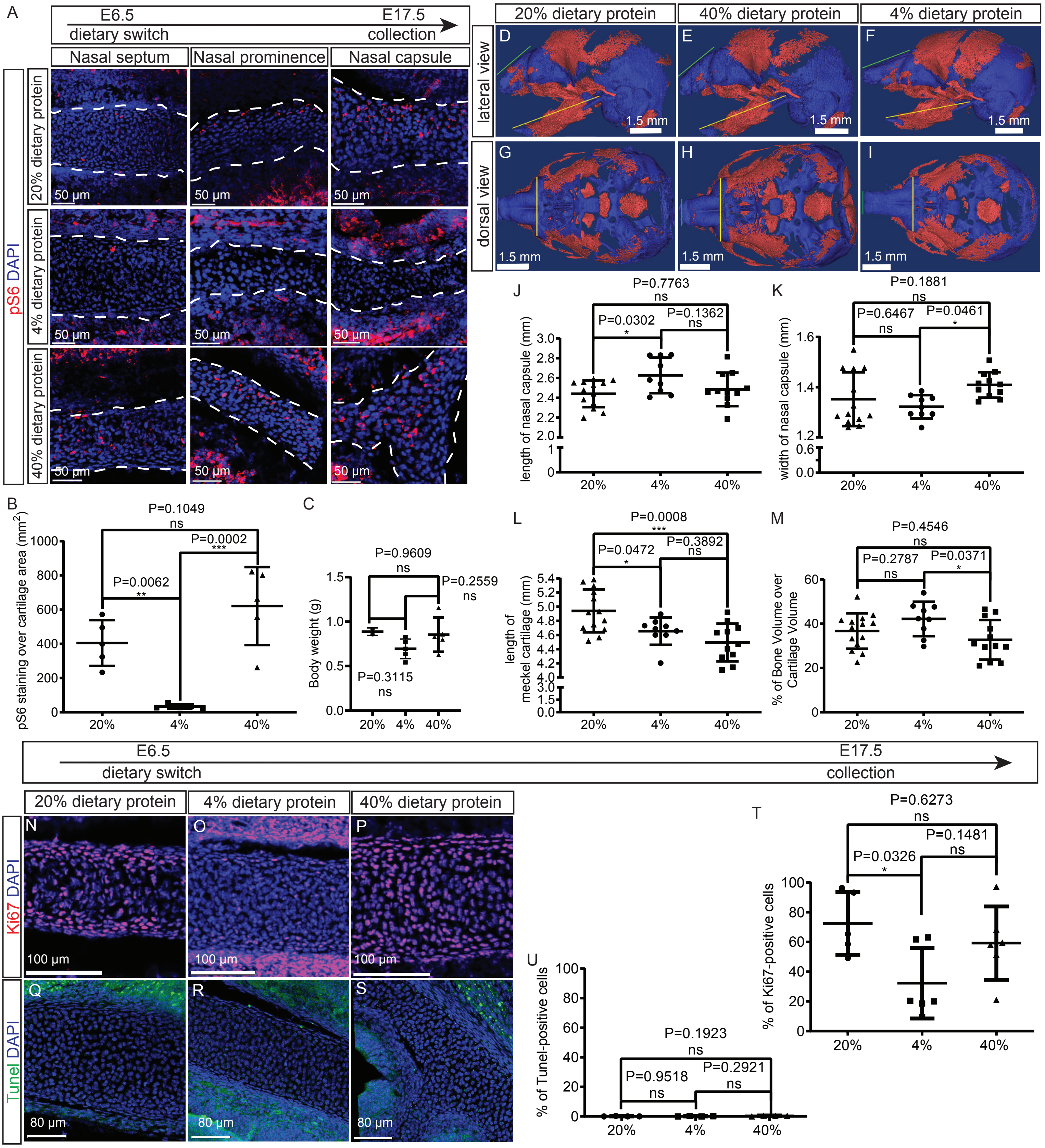

Supplementary Figure 3. *The effect of manipulation of protein levels in maternal diet on craniofacial parameters, mTORC1 activity, cell proliferation and apoptosis in the embryos*

(A-C) Pregnant C57BL/6J dams were placed on isocaloric diets containing either 4%, 20% (control, standard chow=22%) or 40% protein from E6.5 of pregnancy. The level of mTORC1 activity was assessed on the basis of S6 phosphorylation (A) in craniofacial structures of the embryos on E17.5 and quantified (B). The embryos were also weighed on E17.5 (C). n=5 animals in (B). n=2 and 5 animals in (C). (D-M) Reconstruction of the craniofacial structures of embryos on E17.5 employing μCT with contrast enhancement by Hexabrix is shown, with cartilage and mineralized bone in blue and red, respectively (D-I). The green and yellow lines in (D-F) depict the lengths of the nasal capsule and Meckel cartilage, respectively, and these lengths were quantified (J,L). The green and yellow lines in (G-I) depict the width of the nasal capsule, quantified (average of two determinations) in (K). The ratio between bone and cartilage was also quantified (M). n=8, 12 and 13 animals in (J-M). (N-T) The number of proliferating KI67-positive cells (N-P) and apoptotic TUNEL-positive cells (Q-S) was quantified in the cartilage of E17.5 embryos whose mothers consumed diets containing the different levels of protein (T, U). n=5 and 6 animals in (T). n=4 animals in (U). Means ± SD are presented, with individual values also indicated. Statistical analysis was performed by one-way ANOVA followed by Tukey's multiple comparisons test. Source data are provided as a Source Data file.

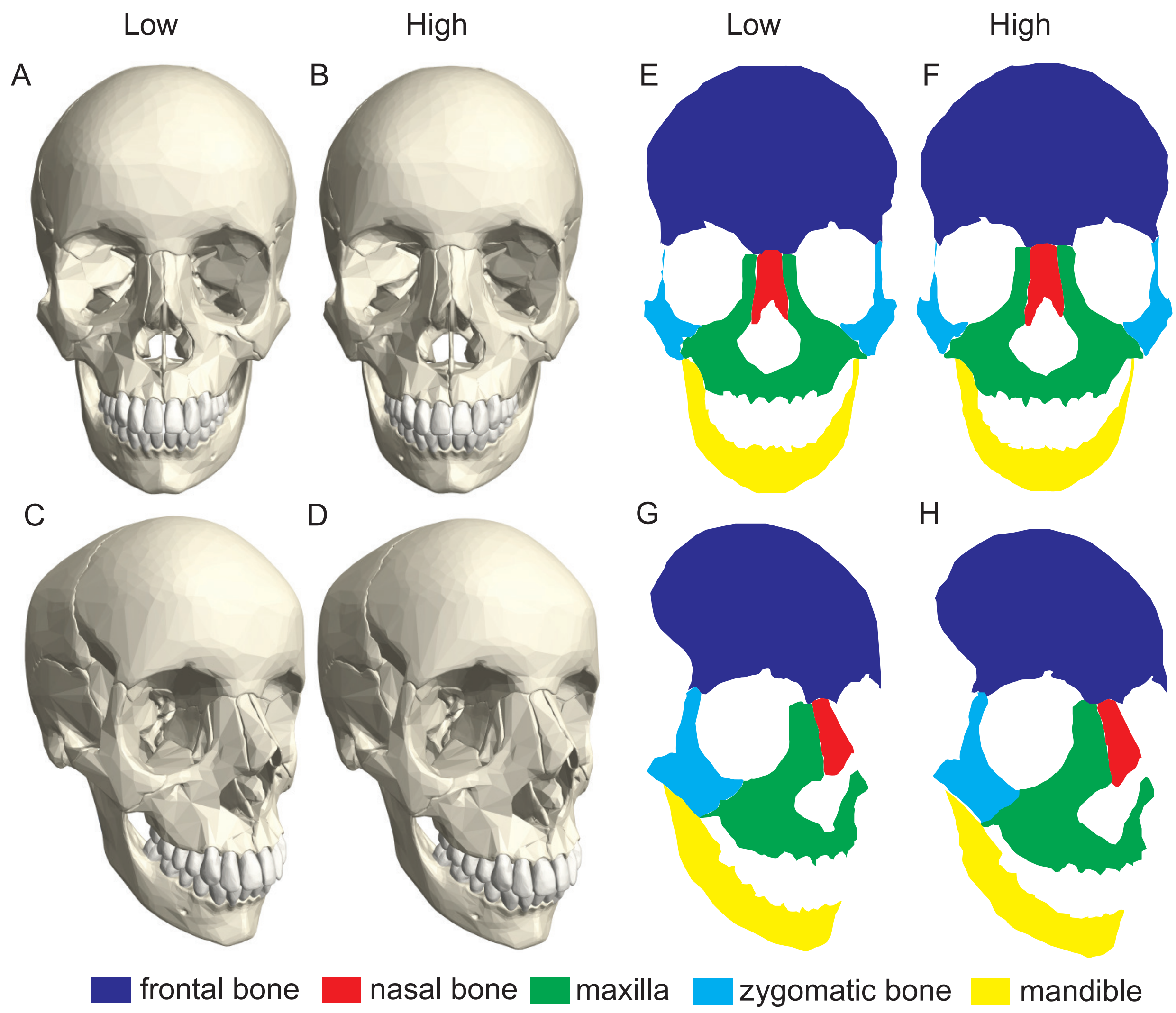

Supplementary Figure 4. *Mathematical prediction of human craniofacial changes upon protein levels in maternal diet, projected from mouse observations*

(A-H) Application of the average craniofacial values for embryos whose dams consumed diets containing low or high levels of protein to a model of the human skull resulted in several structural changes in the craniofacial skeleton. (E-H) depict schematic outlines of the different skeletal elements shown in (A-D), respectively. The algorithm utilized for this conversion is described in the Methods. The original image was obtained from BodyParts3D, © The Database Center for Life Science licensed under CC Attribution-Share Alike 2.1 Japan under CC-BY-SA license https://creativecommons.org/licenses/by-sa/4.0/deed.en
