# Supplementary material for: The level of protein in the maternal murine diet modulates the facial appearance of the offspring via mTORC1 signaling
Source: Nat Commun. 2024 Mar 26;15:2367. doi: 10.1038/s41467-024-46030-3 (PMC10965948; doi:10.1038/s41467-024-46030-3)
Supplement: Supplementary file 3 — Reporting Summary [file 41467_2024_46030_MOESM3_ESM.pdf]

nature portfolio

Corresponding author(s): Andrei S Chagin

Last updated by author(s): Jan 22, 2024

Reporting Summary

Nature Portfolio wishes to improve the reproducibility of the work that we publish. This form provides structure for consistency and transparency in reporting. For further information on Nature Portfolio policies, see our Editorial Policies and the Editorial Policy Checklist.

Statistics

For all statistical analyses, confirm that the following items are present in the figure legend, table legend, main text, or Methods section.

n/a	Confirmed	
☐	☒	The exact sample size (*n*) for each experimental group/condition, given as a discrete number and unit of measurement
☐	☒	A statement on whether measurements were taken from distinct samples or whether the same sample was measured repeatedly
☐	☒	The statistical test(s) used AND whether they are one- or two-sided<br>*Only common tests should be described solely by name; describe more complex techniques in the Methods section.*
☒	☐	A description of all covariates tested
☐	☒	A description of any assumptions or corrections, such as tests of normality and adjustment for multiple comparisons
☐	☒	A full description of the statistical parameters including central tendency (e.g. means) or other basic estimates (e.g. regression coefficient) AND variation (e.g. standard deviation) or associated estimates of uncertainty (e.g. confidence intervals)
☐	☒	For null hypothesis testing, the test statistic (e.g. *F*, *t*, *r*) with confidence intervals, effect sizes, degrees of freedom and *P* value noted<br>*Give P values as exact values whenever suitable.*
☒	☐	For Bayesian analysis, information on the choice of priors and Markov chain Monte Carlo settings
☒	☐	For hierarchical and complex designs, identification of the appropriate level for tests and full reporting of outcomes
☒	☐	Estimates of effect sizes (e.g. Cohen's *d*, Pearson's *r*), indicating how they were calculated

Our web collection on statistics for biologists contains articles on many of the points above.

Software and code

Policy information about availability of computer code

Data collection	Microscopic Images were acquired with an LSM710 confocal microscope. 3D visualization and all quantification were performed utilizing the IMARIS (Bitplane) and ImageJ software. Micro-CT scans were done with GE phoenix v|tome|x L 240 system and NanoTom M (GE Measurement and Control Solutions, Germany) system and analyzed with the DataViewer (Bruker MicroCT, Belgium) and the Avizo (Thermo Fisher Scientific, USA) and VG Studio MAX 3.2 software (Volume Graphics GmbH, Germany).
Data analysis	Data modeling was done with a custom-made code. The code is now deposited here https://zenodo.org/records/10363659 (doi: https://doi.org/10.5281/zenodo.10363659)

For manuscripts utilizing custom algorithms or software that are central to the research but not yet described in published literature, software must be made available to editors and reviewers. We strongly encourage code deposition in a community repository (e.g. GitHub). See the Nature Portfolio guidelines for submitting code & software for further information.

Data

Policy information about availability of data

All manuscripts must include a data availability statement. This statement should provide the following information, where applicable:

- Accession codes, unique identifiers, or web links for publicly available datasets
- A description of any restrictions on data availability
- For clinical datasets or third party data, please ensure that the statement adheres to our policy

The generated datasets are deposited here https://zenodo.org/records/10363659 (doi: https://doi.org/10.5281/zenodo.10363659).
In the study STRING and ENCODE Project Consortium datasets were used.

Research involving human participants, their data, or biological material

Policy information about studies with human participants or human data. See also policy information about sex, gender (identity/presentation), and sexual orientation and race, ethnicity and racism.

Reporting on sex and gender	No sex determination was performed. Both sexes were equally treated.
Reporting on race, ethnicity, or other socially relevant groupings	not applicable
Population characteristics	embryos between 3 and 12 WPC.
Recruitment	All the samples used in the study were non-pathologic, free-will-based abortion-derived.
Ethics oversight	Here we declare that the analysis of human embryos reported here was authorized by the local ethics committee of the Institute of Fundamental Medicine and Biology of Kazan Federal University, that the study design and conduct complied with all relevant regulations regarding the use of human study participants, and was conducted in accordance with the criteria set by the Declaration of Helsinki. the local ethics committee of the Institute of Fundamental Medicine and Biology of Kazan Federal University (No. 8, May 2018)

Note that full information on the approval of the study protocol must also be provided in the manuscript.

Field-specific reporting

Please select the one below that is the best fit for your research. If you are not sure, read the appropriate sections before making your selection.

☒ Life sciences ☐ Behavioural & social sciences ☐ Ecological, evolutionary & environmental sciences

For a reference copy of the document with all sections, see nature.com/documents/nr-reporting-summary-flat.pdf

Life sciences study design

All studies must disclose on these points even when the disclosure is negative.

Sample size	The sample size was chosen based on the expense of data collection and the need to have sufficient statistical power. The null hypothesis was tested with a minimum of 3 (usually 3-7) independent observations (i.e., mice) and if rejected with a statistical power 0.9 or above, the number of observation retained as tested.
Data exclusions	No data were excluded from the analysis.
Replication	All observations were done on samples from different mice (embryos) where each mouse/embryo was considered as an individual observation if not stated otherwise. Mice were collected from at least 2 independent litters, unless otherwise stated. The exact number of observations is indicated in relation to every figure in the corresponding legend. No data were presented in the manuscript, which cannot be reproduced. All the data were included in the analyses.
Randomization	Randomization was performed blindly among animals of the same genotype. Many experiments were not randomized, and the investigators were not blinded to allocation during experiments and outcome assessment because genotyping was required before analysis. All collected human embryos were taken for the analysis.
Blinding	Analysis was performed by a blinded observer wherever possible. Exception was made only for comparing groups with obvious changes in phenotype where the genotyping was required to determine embryos for subsequent analysis.

Reporting for specific materials, systems and methods

We require information from authors about some types of materials, experimental systems and methods used in many studies. Here, indicate whether each material, system or method listed is relevant to your study. If you are not sure if a list item applies to your research, read the appropriate section before selecting a response.

Materials & experimental systems

n/a	Involved in the study
☐	☒ Antibodies
☒	☐ Eukaryotic cell lines
☒	☐ Palaeontology and archaeology
☐	☒ Animals and other organisms
☒	☐ Clinical data
☒	☐ Dual use research of concern
☒	☐ Plants

Methods

n/a	Involved in the study
☒	☐ ChIP-seq
☒	☐ Flow cytometry
☒	☐ MRI-based neuroimaging

Antibodies

Antibodies used

anti-pS6 (Cell Signaling, #2211), anti-SOX9 (Sox9, HPA001758, Sigma Aldrich Inc.), or anti-K67 (Termofisher, MA5-14520)

Validation

for immunohistochemical validation for all antibodies positively-labelled cells have been detected in surrounding tissues with cell type-specific, but not uniform pattern.More detailed information about these antibodies is available on the manufacturers' websites.
anti-pS6 Ab - https://www.cellsignal.com/products/primary-antibodies/phospho-s6-ribosomal-protein-ser235-236-antibody/2211
anti-Sox9 Ab - https://www.sigmaaldrich.com/SE/en/product/sigma/hpa001758
anti-Ki67 Ab - https://www.thermofisher.com/antibody/product/Ki-67-Antibody-clone-SP6-Recombinant-Monoclonal/MA5-14520

Animals and other research organisms

Policy information about studies involving animals; ARRIVE guidelines recommended for reporting animal research, and Sex and Gender in Research

Laboratory animals

Genetically modified mice (mus musculus) were utilized throughout the study. Mice were of a mixed genetic background due to extensive crossing, but with predominance of C57/BL6 background. All animals were housed under a 12:12 hour light/dark cycle with free access to food and water.

Both male and female embryos were employed in approximate ratio 50:50, and no special gender selection was performed. Mice were used from embryonic day E12.5 till E17.5
The following genetic strains were employed in the study:
Col2-CreERt strain, developed by Susan Mackem (NIH)
Rosa26R-Confetti, developed by Hans Clevers (Hubrecht Institute) Tsc1-floxed mice, Jackson Laboratories (JAX#005680)
Raptor-floxed mice, Jackson Laboratories (JAX#013188), Sox10-CreERT2 mice, Jackson Laboratories (JAX#027651)

The Col2a1aBAC:mcherry strain of zebrafish was kindly provided by Prof. Chrissy Hammond (University of Bristol, UK) and analyzed within 120h post-fertilization.

Wild animals

Not used

Reporting on sex

No sex-related selection was applied.

Field-collected samples

no field collected samples were used in the study.

Ethics oversight

All animal experiments were pre-approved by the Stockholm North Ethical Committee and/or Goteborg's Animal Ethical Committee and performed in accordance with the guidelines of the Swedish Animal Agency.

Note that full information on the approval of the study protocol must also be provided in the manuscript.

Plants

Seed stocks

NA

Novel plant genotypes

NA

Authentication

NA
